# Another Law Regulating Dental Practice

**Published:** 1883-07

**Authors:** 


### Meeting of the Board of Examiners.

### Another Law Regulating Dental Practice.

Accompanying this will be found the law regulating the practice of dentistry in the State of Michigan, enacted by its Legislature and sanctioned by the Governor within the past few days.

This is a matter of considerable interest to the public and profession of Michigan.

This State has for a number of years been surrounded by States having such laws, and in which quacks could not prosper, but in Michigan they have found an open and free field, where quackery could flourish without let or hindrance, and large numbers have availed themselves of this opportunity; and, perhaps, no State in the Union has a larger number relatively of incompetent dentists than Michigan, but this state of affairs will undergo rapid changes from this time onward.

The profession of the State, some of them at least, have worked industriously for this law for several years, and for their industry and perseverance deserve success. Prominent among those who were mainly instrumental in finally securing the passage of this law, were Drs. A. T. Metcalf, of Kalamazoo, and G. R. Thomas, of Detroit. But for the untiring labors of these gentlemen, it is doubtful whether the law would have been secured, at the present at least.

The profession of Michigan owe these gentlemen much for their success in this matter.

It was eminently fitting that this law should be enacted. Nearly ten years ago the Legislature established a Dental College in connection with, and under the auspices of the State University at Ann Arbor, and supplied it with all needed facilities for giving a thorough dental education, and it would have been but justice to the public to have passed such a law as this, at that time, thereby saying to those who, after that period, would enter this branch of the healing art, you must prepare yourselves well for this responsible work. But better now than not at all. This law, if properly executed, will work a revolution in the dental profession in the State of Michigan in a few years. The provisions of the law are good; it contains one feature that every such law should have, viz: That of registration. It now behooves the profession of this State to eliminate the debris that has accumulated within her borders, and put the profession in a position that shall nowhere have a superior.

A Bill to Regulate the Practice of Dentistry in the State of Michigan.

Section 1. *The People of the State of Michigan enact*, That it shall hereafter be unlawful for any person to practice dentistry in this State unless such person has received a diploma from the faculty of a reputable dental college duly incorporated under the laws of this or some other State of the United States, or a certificate of qualification from the board of examiners provided for by this act: *Provided,* That the provisions of this section shall in no way apply to or affect any person who is now located and in actual practice in this State.

Sec. 2. Said board of examiners shall be appointed by the Governor of this State, and shall consist of three practical dentists, who shall be regular graduates of a reputable dental college duly incorporated under the laws of this or some other State of the United States, or otherwise possess the necessary qualifications contemplated by this act.

Sec. 3. Each member of this board of examiners shall serve for a term of three years, and until his successor is duly appointed and qualified; except in case of the first board, the members thereof shall serve respectively one, two, and three years, as specified in the appointment of the Governor.

Sec. 4. The board of examiners shall be organized as follows: The member having but one year to serve shall be president of the board; the one having two years shall be treasurer, and the one having three years shall be secretary. The treasurer shall make and file with the Secretary of State a good and sufficient bond to the people of the State of Michigan in the penal sum of one thousand dollars, conditioned that he will well and truly pay over all moneys received by him as such treasurer in compliance with the provisions of this act, and otherwise faithfully discharge the duties of his office.

Sec. 5. The board of examiners shall meet at least once in each year for the purpose of examining applicants after having given personally or by mail thirty days written or printed notice to each practicing dentist in the State who has filed his name and post-office address with the secretary of said board. The said board is authorized to incur all necessary expenses in the prompt and efficient discharge of its duties, and pay the same with any moneys in the hands of its treasurer.

Sec. 6. Each member of said board shall qualify by taking the oath of office prescribed by the constitution of this State, and filing the same with the Secretary of State before entering upon the duties of his office. Should a vacancy occur in said board, the Governor of this State shall fill the same by appointment.

SEC. 7. Any member of said board of examiners may, when the board is not in session, examine applicants, and in case any applicant is found competent grant a license to him to practice dentistry in this State until the next meeting of the said board and no longer, upon the payment of the sum of three dollars: *Provided*, No member of the said board shall grant a license to one who has been rejected on an examination by the board.

SEC. 8. Should any member of said board be unable to attend at the meeting of the board for the examination of applicants, he may appoint in writing, a substitute, who shall have the same power on the examination that the member appointing him would have if present: *Provided*, Such substitute be a person eligible to be a member of said board within the provisions of this act: *And provided further*, That the appointment of such substitute be by and with the written consent of the other members of the board.

SEC. 9. Each applicant shall, on receipt of a license to practice, pay into the treasury of the board the sum of ten dollars, which shall constitute a fund to defray the expenses of the board; and each member of the board shall receive therefrom the sum of three dollars per day for services rendered as such examiner. The said board shall keep a list of the names of all persons to whom licenses have been granted under the provisions of this act, and also of all persons practicing dentistry in this State in a book provided for that purpose with the names arranged in alphabetical order.

SEC. 10. Any sum in excess of one hundred dollars which, under the provisions of this act, may accumulate in the treasury of said board, shall be paid by the treasurer thereof into the treasury of this State.

SEC. 11. Each person now engaged in the practice of dentistry in this State shall within ninety days after this act takes effect send an affidavit to the secretary of said board setting forth his name, place of business, postoffice address, the length of time he has been engaged in practice in this State, and if a graduate of a dental college state the name of the same, and also pay the treasurer of said board the sum of twenty-five cents, and on failure to comply with the provisions of this section he shall be required to appear and be examined by said board.

Sec. 12. Any person who shall practice dentistry in this State in violation of the provisions of this act shall be deemed guilty of a misdemeanor, and upon conviction thereof shall be fined not less than twenty-five dollars nor more than one hundred dollars, or sentenced to imprisonment in the county jail for a period not exceeding ninety days, or both fine and imprisonment in the discretion of the court: *Provided*, That nothing in this act shall be construed so as to interfere with physicians and surgeons in their practice as such.

